# Amplified Cell Cycle Genes Identified in High-Grade Serous Ovarian Cancer

**DOI:** 10.3390/cancers16162783

**Published:** 2024-08-07

**Authors:** Karthik Balakrishnan, Yuanhong Chen, Jixin Dong

**Affiliations:** Eppley Institute for Research in Cancer and Allied Diseases, Fred & Pamela Buffett Cancer Center, University of Nebraska Medical Center, Omaha, NE 68198, USA; kbalakrishnan@unmc.edu (K.B.); cheny@unmc.edu (Y.C.)

**Keywords:** ovarian cancer, serous subtype, differentially expressed gene, amplification, cancer recurrence

## Abstract

**Simple Summary:**

The current investigation identifies differentially expressed genes that specifically influence the serous subtype of ovarian cancer. This subtype accounts for around three-quarters of ovarian cancer cases. To identify these genes, transcriptomic profiles of serous ovarian cancer and non-cancerous tissue samples were extracted from the Gene Expression Omnibus. Differentially expressed genes were derived using GEO2R tool analyses; genes consistently found among upregulated genes in these profiles were considered to be a serous gene set. Next, the serous gene set was examined for its ontological function using the Molecular Signatures Database and its mutational impact on the gene expression profile of high-grade serous ovarian (HGSO) adenocarcinoma. Results showed that 26 genes are amplified in over 5% of HGSO cancer patients, and many of these amplified genes are related to the cell cycle. These cell cycle-related genes were also identified as being involved in the recurrence of serous ovarian cancer. Overall, this study identifies genes that are potential prognostic markers for serous ovarian cancer.

**Abstract:**

The objective of this study was to identify differentially expressed genes and their potential influence on the carcinogenesis of serous-type ovarian cancer tumors. Serous cancer is an epithelial ovarian cancer subtype and is the most common type of ovarian cancer. Transcriptomic profiles of serous cancer and non-cancerous datasets were obtained from the Gene Expression Omnibus (GEO-NCBI). Differentially expressed genes were then derived from those profiles; the identified genes were consistently upregulated in three or more transcriptomic profiles. These genes were considered as the serous ovarian cancer gene set for further study. The serous gene set derived from the transcriptomic profiles was then evaluated for ontological functional analysis using the Molecular Signatures Database. Next, we examined the mutational impact of this serous gene set on the transcriptomic profile of high-grade serous ovarian (HGSO) adenocarcinoma using the cBioPortal database. Results from OncoPrint revealed that 26 genes were amplified in more than 5% of HGSO cancer patients. Interestingly, several of these genes are involved in cell cycle processes, including genes ATPase family AAA domain containing 2 (ATAD2), recQ-like helicase 4 (RECQL4), cyclin E1 (CCNE1), anti-silencing function 1B histone chaperone (ASF1B), ribonuclease H2 subunit A (RNASEH2A), structural maintenance of chromosome 4 (SMC4), cell division cycle associated 20 (CDC20), and cell division cycle associated 8 (CDCA8). The receiver operating characteristic (ROC) curve results also revealed higher specificity and sensitivity for this subtype of tumors. Furthermore, these genes may affect the recurrence of serous ovarian carcinogenesis. Overall, our analytical study identifies cell cycle-related genes that can potentially be targeted as diagnostic and prognostic markers for serous ovarian cancer.

## 1. Introduction

Ovarian cancer is the fifth most common cancer among women worldwide [1,2]. Every year, ovarian cancer results in 150,000 deaths globally. In the United States, 19,710 new ovarian cancer cases and resultant 13,270 deaths occur annually [3]. Unfortunately, due to the lack of early diagnostic markers, most ovarian cancer patients are identified after the cancer has metastasized [4].

Clinically, ovarian cancers can be classified into the following five main histological subtypes: endometrioid ovarian cancer, high-grade serous ovarian (HGSO) cancer, mucinous ovarian cancer, clear-cell ovarian cancer, and low-grade serous ovarian carcinoma [5]. HGSO adenocarcinoma is the most common ovarian cancer [6], and due to its dismal prognosis, it accounts for 70–80% of ovarian cancer mortalities [7]. In addition, metastatic features of this cancer differ from that of many other human cancers in that it has been found that the cells are released from the ovaries and fallopian tubes into the peritoneal space, where they congregate and move to other organs [8,9]. Understanding the early pathogenesis of ovarian malignancies is key to locating and identifying novel gene markers for early diagnosis, as well as for paving the way for efficient preventive therapy strategies and targeted effective treatments.

The present study uses integrative functional genomic strategies to identify differentially expressed genes in serous ovarian cancer and non-cancerous tissue samples comprising five different mRNA expression profiles. The differentially expressed genes were identified using these profiles of serous-subtype-specific ovarian tumors. Moreover, the genes identified were examined via mutation analysis, receiver operating characteristic (ROC) curves, and overall survival curve plots to further confirm this subtype-specific gene set is involved in ovarian carcinogenesis. Interestingly, eight genes involved in the cell cycle were found to be significantly amplified and dysregulated in HGSO cancer patient samples. The findings from this study can be applied to identify prognostic markers and develop therapeutic targets for serous-subtype-specific ovarian cancer tumors.

## 2. Materials and Methods

### 2.1. Collection of Expression Profiles

The Gene Expression Omnibus (GEO), a publicly available genome-wide database by the National Center for Biotechnology Information, was utilized to obtain mRNA expression profiles [10]. The following non-cancerous and serous cancer subtypes that comprise ovarian cancer profiles were gathered and utilized in this study: GSE10971, GSE36668, GSE14407, GSE12470, GSE6008, and recurrence profile GSE44104. Next, normalized series matrix files were obtained or raw data. CEL files were collected from GEO. Then R software (4.4.1) was used for MAS5/RMA normalization using the Affy Package [11]. The appropriate platform annotation data were used to map the probes to distinct gene symbols. Gene expression values with numerous probes were averaged and utilized in subsequent studies.

### 2.2. Differential Gene Expression Analysis

The following five mRNA expression profiles were used in this study: GSE10971, GSE36668, GSE14407, GSE12470, and GSE6008. Differential gene expression analysis was conducted using GEO2R [12,13]. Genes that revealed upregulated or downregulated expression with a fold change of ≥2 or ≤−2 and a *p*-value of ≤0.05 were considered statistically significant and were used for further examination. Genes that were consistently upregulated across the mRNA expression profiles were identified using the Bioinformatics and Evolutionary Genomics Venn diagram webtool (https://bioinformatics.psb.ugent.be/webtools/Venn/ (accessed on 1 April 2024).

### 2.3. Hierarchical Clustering and Visualization

Hierarchical clustering is a statistical method used to locate gene expression patterns in specific biological or experimental conditions [14]. Genes with identical expression patterns can reveal their associated functionality. As such, dChip software (2011.12) was used for hierarchical cluster analysis [15,16,17]. A heatmap representation was used to show the hierarchical clustering of genes and samples. The upregulated genes were further examined by the Molecular Signatures Database (MSigDB) of gene set enrichment analysis (GSEA) [18]. The identified gene set was studied with distant regulatory elements of co-expressed genes (DiRE) for identifying the regulatory transcription factors [19]. Additionally, gene expression profiling interactive analysis 2 (GEPIA2) was used as a web resource for gene expression analysis comparing ovarian tumor tissue samples with normal tissue samples [20].

### 2.4. Mutation Analysis

An integrated functional genomics study was carried out in the RNA sequencing profile of HGSO cancer using cBioPortal (https://www.cbioportal.org/ (accessed on 5 April 2024), a publicly accessible resource to examine tumor genomics and transcriptomics [21]. This database was used for the large-scale examination of the transcriptome and genomic data and to visually depict tumor changes. The serous gene set was analyzed for the impact of its mutation levels on the carcinogenesis of the serous ovarian cancer subtype.

### 2.5. ROC Curve

Ovarian malignancies are associated with several tumor subtypes. The cell cycle processes involving genes ATAD2, RECQL4, CCNE1, ASF1B, RNASEH2A, and SMC4, gene expression values were evaluated in the following profiles: GSE10971, GSE36668, GSE14407, and GSE12470. The MedCalc tool was utilized to plot the receiver operating characteristic (ROC) curves [22,23]. 

### 2.6. Survival Analysis

The overall survival curve was obtained using the Kaplan–Meier plot [24,25,26]. The Kaplan–Meier plot tools were used together with data analysis of clinical features and mortality rates to examine the potential effect of specific gene expression on the survival pattern of patients with serous subtypes of ovarian cancer. The plot analysis employed the following eight genes: ASF1B, ATAD2, CCNE1, CDC20, CDCA8, RECQL4, RNASEH2A, and SMC4. The *p*-values were obtained from the log-rank test [27].

### 2.7. Western Blotting Analysis

Non-transformed cells of human ovarian surface epithelial T80 cell lines (Hose-T80) and high-grade serous ovarian adenocarcinoma (HGSOC) cell lines (OVCAR4, OVCAR8, and OVCAR3) were maintained in DMEM/ RPMI media containing 10% FBS and supplemented with 100 units/mL penicillin and 100 µg/mL streptomycin at 37 °C in a 5% CO_2_ incubator. The proteins were run on 8% or 12% SDS polyacrylamide gels and transferred onto PVDF membranes. Western blotting analysis was done as previously defined [28]. Monoclonal anti-ASF1B, CCNE1, SMC4, CDC20 antibodies were from Cell Signaling Technology (Danvers, MA, USA) and used at 1:1000 dilutions. Rabbit anti-RNASEH2A antibodies were from Fortis Life Sciences/Bethyl Laboratories (1:1000 dilution) (Dallas, TX, USA). Monoclonal anti-CDCA8 (Borealin, 1:250), RECQL4 (1:250), β-actin (1:2000) antibodies were purchased from Santa Cruz Biotechnology (Dallas, TX, USA).

## 3. Results

### 3.1. Identification of Differentially Expressed Genes in Serous-Type Ovarian Cancer

**Figure 1:**
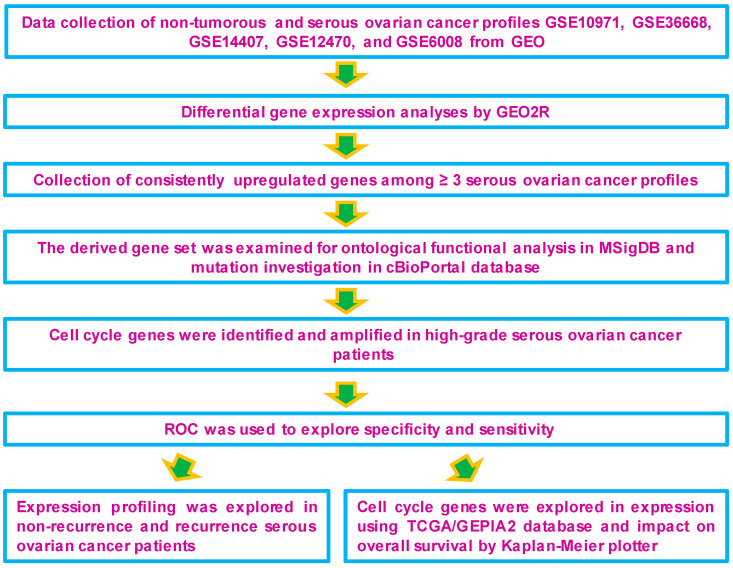
The overall workflow for identifying and validating differentially expressed and amplified cell cycle genes in serous-subtype-specific ovarian carcinogenesis.

**Figure 2:**
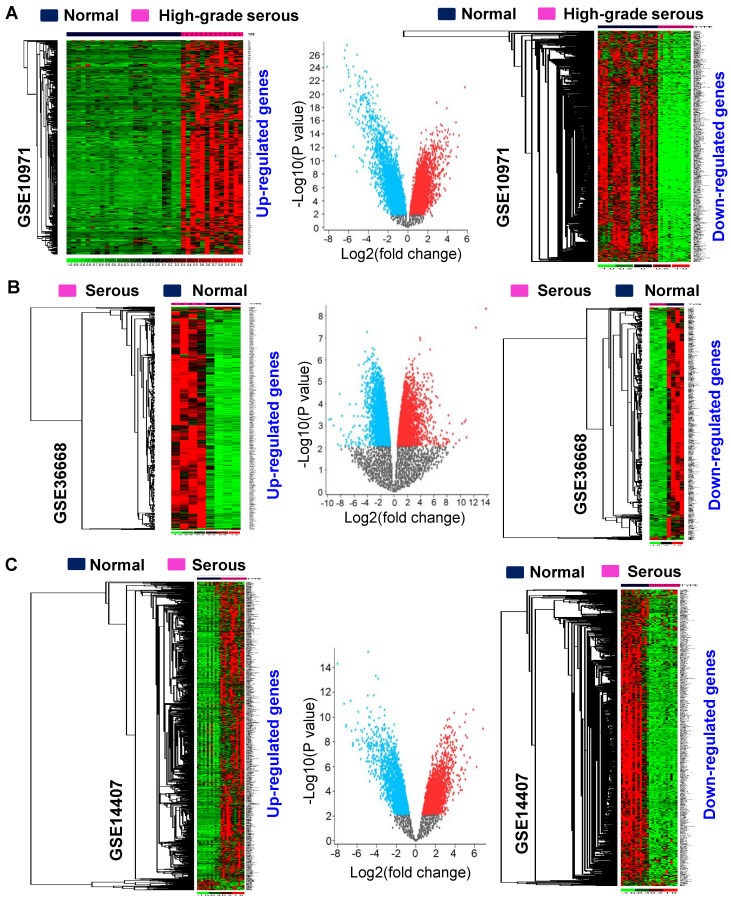
The identified differentially expressed gene expression patterns were confirmed in the corresponding profiles. (**A**–**C**) The following non-tumors and serous subtypes comprising ovarian cancer profiles were used: GSE10971 (**A**), GSE36668 (**B**), and GSE14407 (**C**). The heatmap shows the serous-subtype-specific expressions of upregulated and downregulated genes with the corresponding volcano plot for those profiles.

The objective of this study was to identify genes that are differentially expressed during the carcinogenesis of serous-type ovarian cancer tumors. The overall workflow and validation strategies used to identify differentially expressed genes in serous-subtype-specific ovarian cancer are shown in (Figure 1). The following five non-tumorous and serous subtypes containing mRNA expression profiles were collected from GEO and used for further analysis: GSE10971, GSE36668, GSE14407, GSE12470, and GSE6008. Differentially expressed genes were derived using the GEO2R tool for these mRNA expression profiles. The genes with a cutoff fold-change value ≥ 2 or ≤−2 with significant *p*-values (*p* < 0.05) were considered as upregulated or downregulated in the study (Table S1). These gene expression patterns were further examined with their corresponding profiles. The heatmap displays the serous type-specific expression of differentially expressed genes and the corresponding volcano plots (Figure 2A–C; Figure S1A,B). Thus, serous-subtype-specific differentially expressed genes were identified and used for further study. 

### 3.2. Identification of Consistently Upregulated Genes in Serous-Type Ovarian Tumors

**Figure 3:**
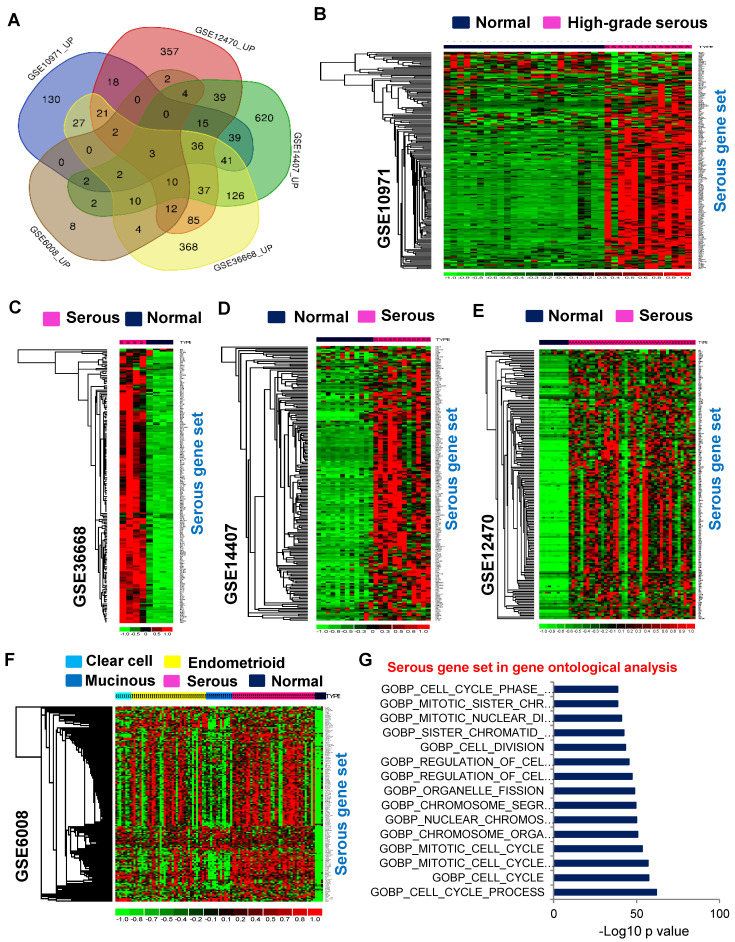
The upregulated genes of the serous cancer subtype were derived from the following five distinct expression profiles and used for further study: GSE10971, GSE36668, GSE14407, GSE12470, and GSE6008. (**A**) The Venn diagram displays the upregulated genes of the five gene expression profiles. The genes consistently found in three or more datasets were considered a serous gene set. (**B**–**F**) The serous gene set was expressed exclusively in serous-type ovarian tumors. (**G**) Ontological functional analysis was performed for the serous gene set using the Molecular Signatures Database of GSEA.

**Figure 4:**
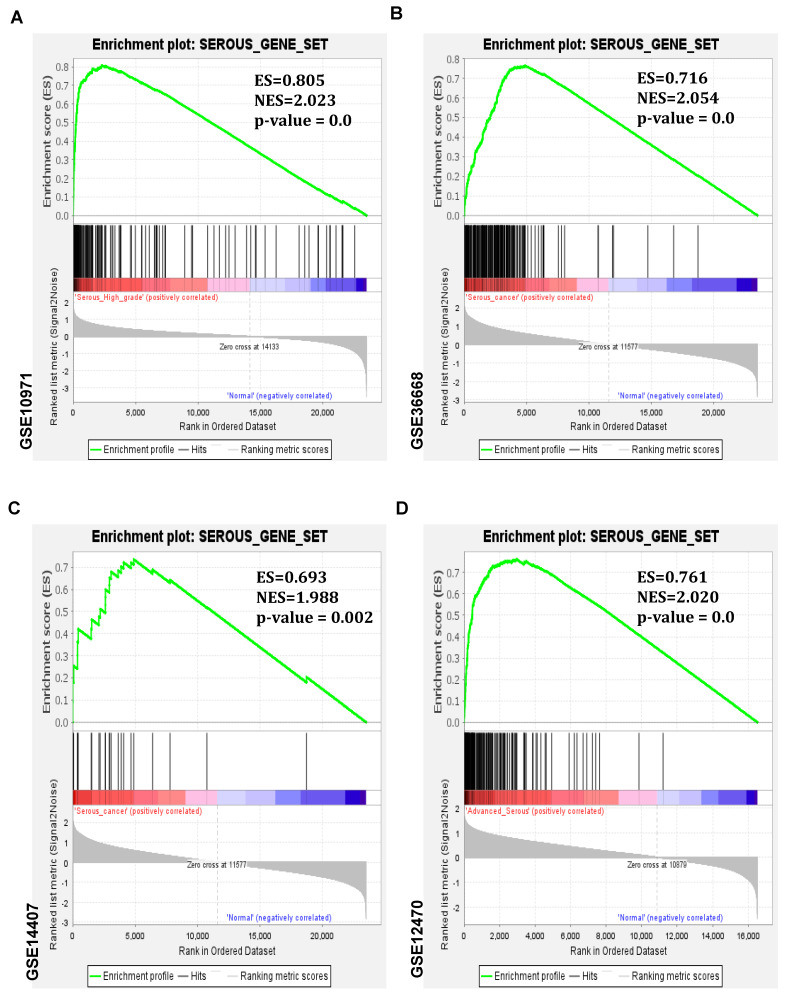
The gene-set enrichment analysis of the following serous-type ovarian cancer mRNA expression profiles was investigated for further confirmation: GSE10971, GSE36668, GSE14407, and GSE12470. (**A**–**D**) The results revealed higher enrichment scores (ES) for serous-subtype-specific ovarian tumors.

The Venn diagram was utilized to identify common genes among the upregulated genes (Figure 3A). In this study, the genes that were consistently found elevated in three or more profiles of the upregulated genes were categorized as a serous gene set (a total of 195 genes) (Table S2). The serous gene set is more highly expressed in serous-type ovarian tumors than in non-tumorous samples of the corresponding mRNA expression profiles (Figure 3B–F). The serous gene set was also investigated in the Molecular Signatures Database (MSigDB) for ontological functional analysis. Results showed these genes play critical roles during serous-type ovarian carcinogenesis through the following cellular processes: cell cycle, chromosomal segregation, and cell division regulation (Figure 3G). The serous gene set was further explored through pathway enrichment analysis in MSigDB. It revealed that these genes are mainly involved in cell cycle-mediated pathway processes (Figure S2A). Additionally, the gene set was examined with distant regulatory elements of co-expressed genes (DiRE) to identify regulatory transcription factors for these genes. The top 10 transcription factors NCX, E2F1DP1, E2F4DP2, VMYB, E2F1DP1RB, STAT1, RFX1, E2F1DP2, PBX1, and PEBP were identified as crucial regulators of expression of the serous gene set (Figure S2B). Similarly, a serous downregulated gene set was derived from the five profiles (Table S3) and was explored by functional ontological analysis using MSigDB. It showed that the downregulated set was involved in negative RNA transcriptional biosynthesis and regulation, muscle tissue structural development, and differentiation in serous ovarian cancer (Figure S2C). In addition, gene set enrichment analysis (GSEA) was employed for additional confirmation. The serous gene set was found to have greater enrichment scores for serous-subtype-specific ovarian cancer (Figure 4A–D). Together, these analyses suggest the genes identified are integral in the pathological features of serous-type ovarian carcinogenesis.

### 3.3. Serous Gene Set Significantly Amplified in HGSO Adenocarcinoma

**Figure 5:**
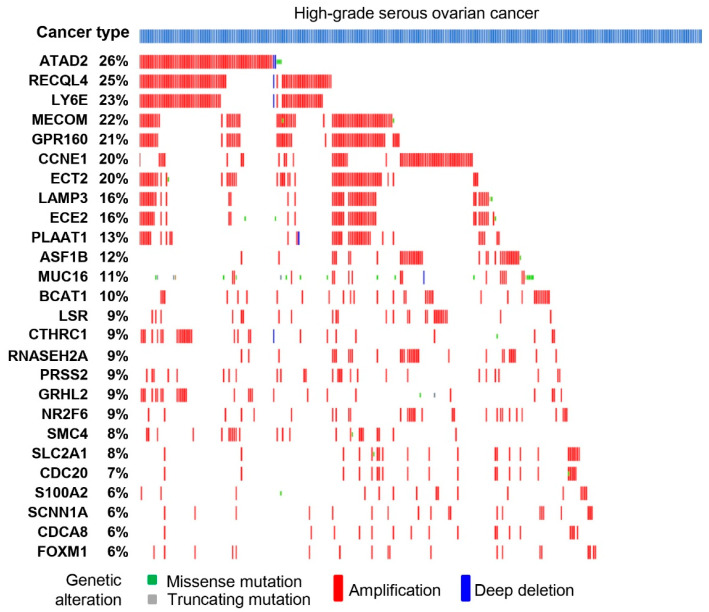
The serous gene set was evaluated for its mutation impact on HGSO cancer profiles. OncoPrint results showed that many cell cycle genes were amplified significantly in more than 5% of samples from HGSO cancer patients.

The serous gene set was further assessed at the mutation level based on the HGSO cancer profile using the cBioPortal database. Among the serous gene set, OncoPrint results showed the following 26 genes were amplified in more than 5% of HGSO adenocarcinoma patient samples: ATPase family AAA domain containing 2 (ATAD2), recQ-like helicase 4 (RECQL4), LY6E, MECOM, GPR160, cyclin E1 (CCNE1), ECT2, LAMP3, ECE2, PLAAT1, anti-silencing function 1B histone chaperone (ASF1B), MUC16, BCAT1, LSR, CTHRC1, ribonuclease H2 subunit A (RNASEH2A), PRSS2, GRHL2, NR2F6, structural maintenance of chromosomes 4 (SMC4), SLC2A1, cell division cycle associated 20 (CDC20), S100A2, SCNN1A, cell division cycle associated 8 (CDCA8), and FOXM1. Interestingly, among these genes, the following eight cell-cycle-related genes were notably amplified in these patient samples: ATAD2, RECQL4, CCNE1, ASF1B, RNASEH2A, SMC4, CDC20, and CDCA8 (Figure 5). Apart from cell cycle genes, the following metabolic genes were also remarkably amplified: PLAAT1, BCAT1, LSR, and SLC2A1 (Figure 5).

### 3.4. Identification of Cell Cycle Genes as Promising Prognostic Markers for Serous-Type Ovarian Cancer

**Figure 6:**
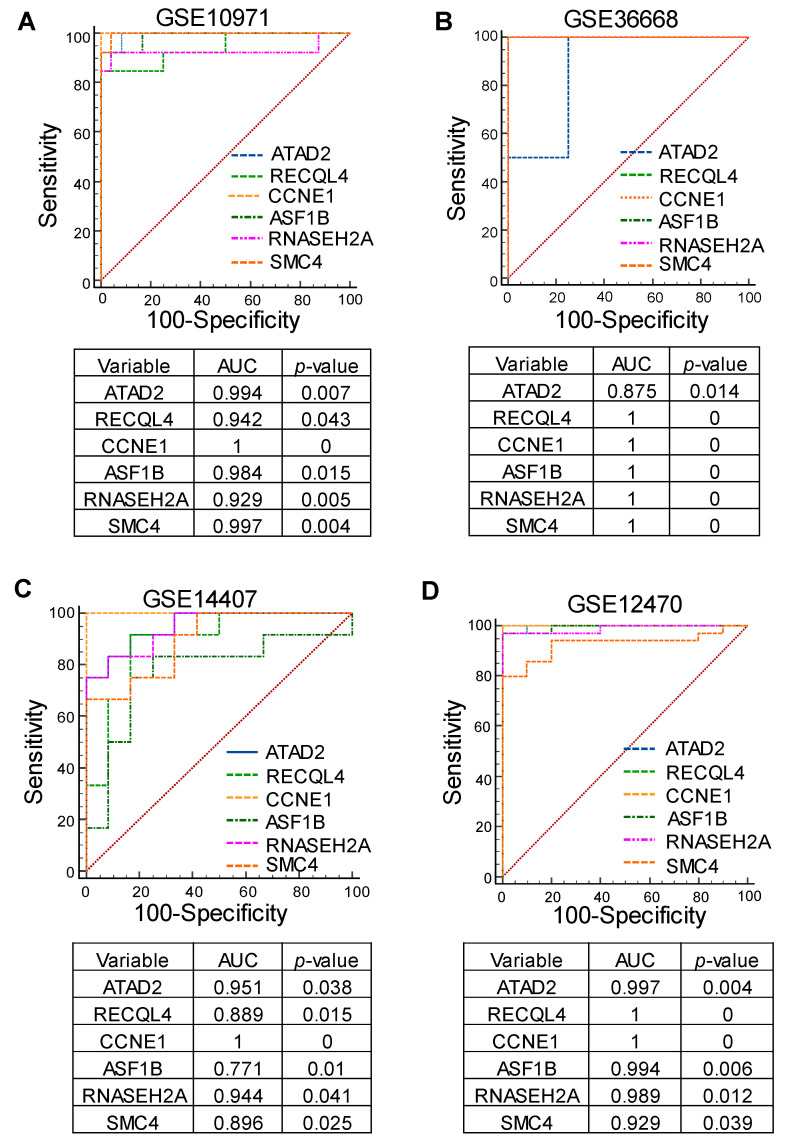
The receiver operating characteristic (ROC) curve-based investigation was accomplished for the following top cell cycle-involved genes: ATAD2, RECQL4, CCNE1, ASF1B, RNASEH2A, and SMC4 expression in profiles GSE10971, GSE36668, GSE14407, and GSE12470. (**A**–**D**) The ROC curve of these genes can predict the serous subtype with greater sensitivity and specificity. The areas under curve (AUC) and *p*-values show steeply elevated values for those genes.

A receiver operating characteristic (ROC) curve-based examination was performed for ATAD2, RECQL4, CCNE1, ASF1B, RNASEH2A, and SMC4 mRNA expression in the following gene expression profiles: GSE10971, GSE36668, GSE14407, and GSE12470. As shown, the ROC curve result can predict the serous subtype with greater sensitivity and specificity based on the remarkably higher values of the areas under the curve (AUC) and more significant *p*-values (Figure 6A–D).

**Figure 7:**
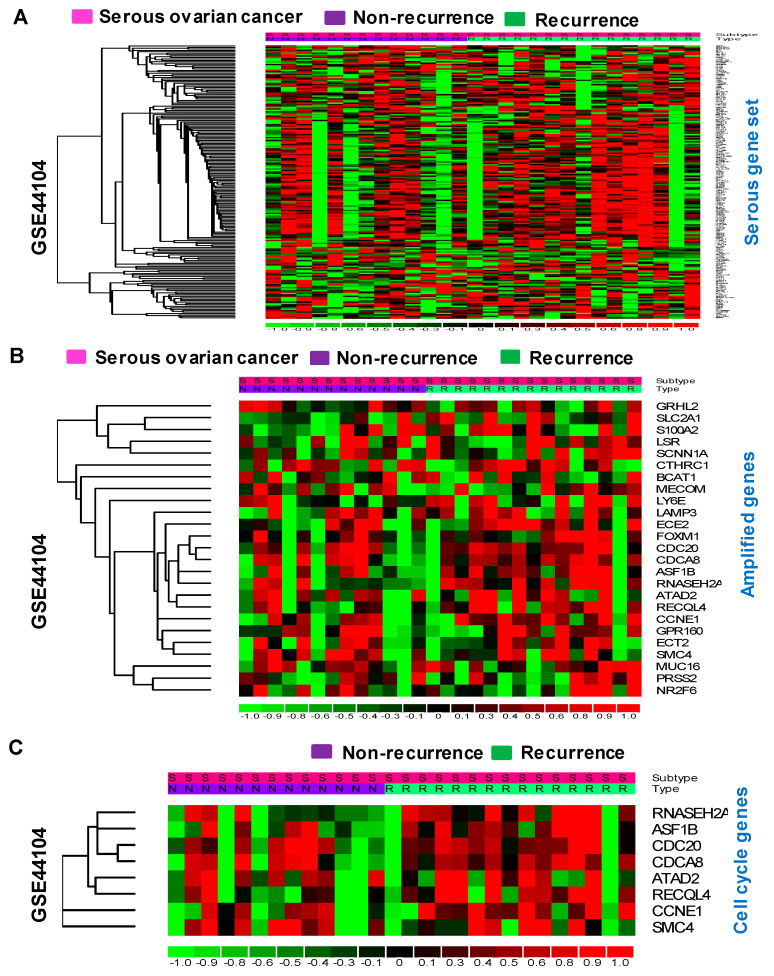
Expression of the serous gene set influences the recurrence of serous ovarian cancer. (**A**,**B**) The serous gene set and amplified genes have a slightly greater expression pattern in recurrent serous samples than in non-recurrent tumors in the GSE44104 dataset. (**C**) The newly identified cell-cycle-involved genes are highly expressed in recurrent serous tumors.

The expression patterns of the serous gene set were also analyzed in recurrent and non-recurrent tumors. The serous gene set and amplified genes were found to have slightly greater expression in recurrent serous tumor samples compared to non-recurrent serous tumors (Figure 7A,B). Likewise, these newly identified cell cycle genes were found to be more intensely expressed in recurrent serous tumors than in non-recurrent serous tumors in ovarian cancer patients (Figure 7C).

**Figure 8:**
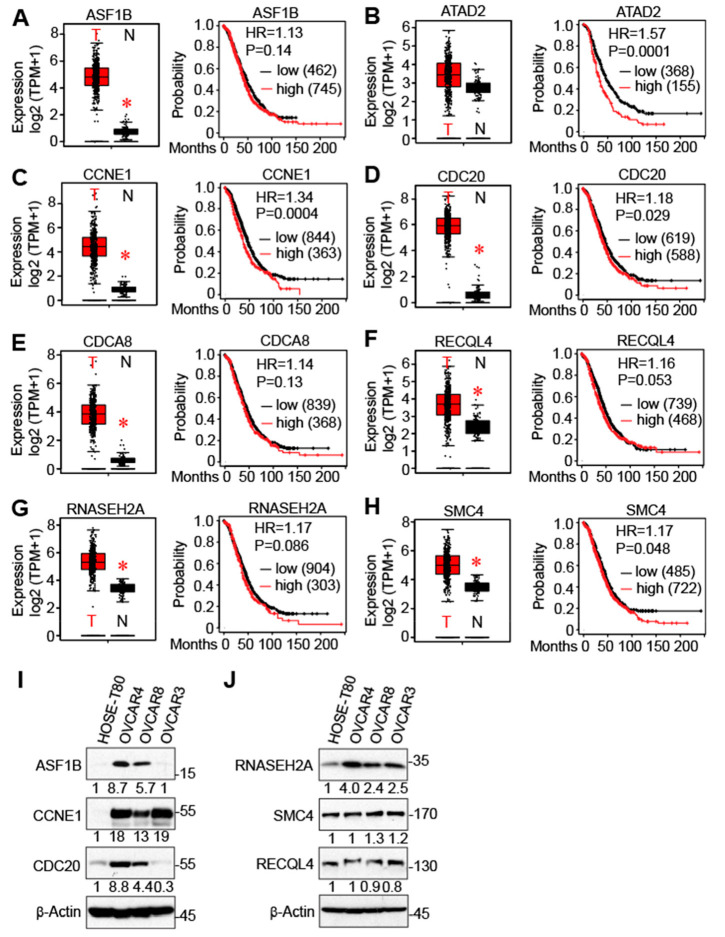
The expression levels of cell cycle genes and associated clinical outcomes (overall survival, OS). The GEPIA2 online tool (using the TCGA database) and Kaplan–Meier (KM) survival curve plots were used for gene expression and OS analysis, respectively (**A**–**H**). T: tumor (*n* = 426); N: normal (*n* = 88). TPM: transcripts per million. *: *p* < 0.01. The log-rank test method was used to identify these *p*-values. (**I**,**J**) Western blot analysis with the indicated antibodies in non-transformed (HOSE-T80) and ovarian cancer cells (OVCAR4, OVCAR8, and OVCAR3). Numbers below each blot are relative intensity normalized to actin. The uncropped blots are shown in Figure S3. Analysis was performed using Image J software (version 1.54).

The expression levels of cell-cycle genes and associated clinical outcomes were determined using the TCGA database and Kaplan–Meier survival curve plots. Except for ATAD2, expression of other genes (ASF1B, CCNE1, CDC20, CDCA8, RECQL4, RNASEH2A, and SMC4) was upregulated in ovarian tumors compared to non-cancerous tissue (Figure 8A–H). Furthermore, we confirmed that protein levels of ASF1B, CCNE1, CDC20, and RNASEH2A were significantly increased in ovarian cancer cells compared with non-transformed ovarian cells (Figure 8I,J). RECQL4 and SMC4 expression is not altered in these cells (Figure 8J). CDCA8 protein was not detectable under these conditions. Additionally, the Kaplan–Meier plot results revealed that increased expression of ATAD2, CCNE1, CDC20, and SMC4 was associated with poor survival in serous ovarian cancer patients (*p* < 0.05). Thus, results indicate these genes are potential prognostic markers for serous-type ovarian tumors.

## 4. Discussion

The serous cancer subtype is the most prevalent histological subtype of ovarian cancer. Serous cancer accounts for 70–80% of ovarian cancer fatalities and has a dismal five-year survival rate [29]. While surgery and other treatment techniques have been developed, prognosis and treatment for ovarian cancer are at a tremendous disadvantage due to a lack of diagnostic identifiers and resultant late diagnoses [30]. Most ovarian cancer cases are detected in women over 75 years of age and in later stages when the cancer cells have developed higher grades and have spread to other locations in the body [31]. Therefore, screening and identifying early novel diagnostic markers is necessary to understand and counteract the underlying pathophysiological mechanisms. In the present study, we investigated the existing gene expression profiles using genomic approaches to find the prognosis-related critical genes for early detection of ovarian cancer with the serous subtype. Our results show that genes from the serous gene set are implicated in ontological functional prominent features in serous tumors, including cell cycle, chromosome segregation, and cell division. 

Earlier genomic investigations of serous ovarian cancer have revealed that rather than containing conventional activating oncogenic mutations, serous ovarian cancer has a highly complicated genomic landscape with notable structural genomic variations and significant copy number aberrations [32]. Several vital genes can acquire gain of function mutations due to the genomic copy number alterations [33]. For example, the mutations in BRCA1 and BRCA2 in serous ovarian cancer were identified as primary cancer drivers, along with mutations in the tumor-suppressor genes like TP53, NF1, RB1, and PTEN [34,35]. The point mutations have been found to be dispersed across the gene, primarily denoting changes that result in loss of function [36]. Due to loss-of-function mutations, TP53 expression is reduced in almost all human malignancies. In addition to individual gene mutations, Hippo signaling pathway genes are significantly amplified and implicated in the carcinogenesis of the serous subtype of ovarian tumors [37].

The study herein identifies serous gene sets that are significantly amplified in HGSO adenocarcinoma patients. The copy number gain of the CCNE1 gene is present in 14% of cases and was mutually exclusive with numerous other recurrent genomic BRCA1/2 mutations [38]. Amplification of the CCNE1 gene is a crucial regulator of the G1/S transition. Likewise, CCNE1 was found to be amplified in serous ovarian cancer patients who had reduced response to chemotherapy and poor survival [39]. The present results also exhibit that among the amplified genes, the following eight genes involved in the cell cycle were found to be amplified in more than 5% of HGSO adenocarcinoma patients: ATAD2, ASF1B, CCNE1, CDC20, CDCA8, RECQL4, RNASEH2A, and SMC4. Moreover, the increased expression of these genes was associated with poor survival. Thus, our findings suggest that these cell-cycle-related genes are potential prognostic markers for serous subtypes of ovarian tumors and might be used for targeted treatments.

## 5. Conclusions

This study identifies differentially expressed genes in serous-subtype-specific ovarian cancer. The expression patterns of the differentially expressed genes were further reconfirmed by GSEA, ROC curve exploration, and by examining the mutational impact on HGSO adenocarcinoma. Among the differentially expressed genes, genes involved in the cell cycle were significantly amplified in HGSO cancer patients. Thus, this study suggests potential prognostic genetic markers and therapeutic targets in serous-subtype-specific ovarian cancer.

## Data Availability

The original data presented in the study are in a publicly accessible repository, and the data presented in this study are available upon request from the corresponding author for valuable reasons.

## Supplementary Materials

The following supporting information can be downloaded at: https://www.mdpi.com/article/10.3390/cancers16162783/s1, Figure S1. The differentially expressed genes in other serous subtypes in GSE12470 and GSE6008 datasets; Figure S2. The derived serous gene set was further analyzed; Figure S3. Uncropped Western Blot images from Figure 8; Table S1. The list of the differentially expressed genes from the 5 datasets; Table S2. The list of genes commonly upregulated in 3 or more profiles among the 5 datasets used in the study; Table S3. The list of genes commonly downregulated in 3 or more profiles among the 5 datasets used in the study.
