# Supplementary material for: Amplified Cell Cycle Genes Identified in High-Grade Serous Ovarian Cancer
Source: Cancers (Basel). 2024 Aug 7;16(16):2783. doi: 10.3390/cancers16162783 (PMC11352846; doi:10.3390/cancers16162783)
Supplement: Supplementary file 1 [file cancers-16-02783-s001.zip › cancers-3071607-supplementary.docx]

Supplementary Materials: Amplified Cell Cycle Genes
Identified in High-Grade Serous Ovarian Cancer

Karthik Balakrishnan, Yuanhong Chen and Jixin Dong


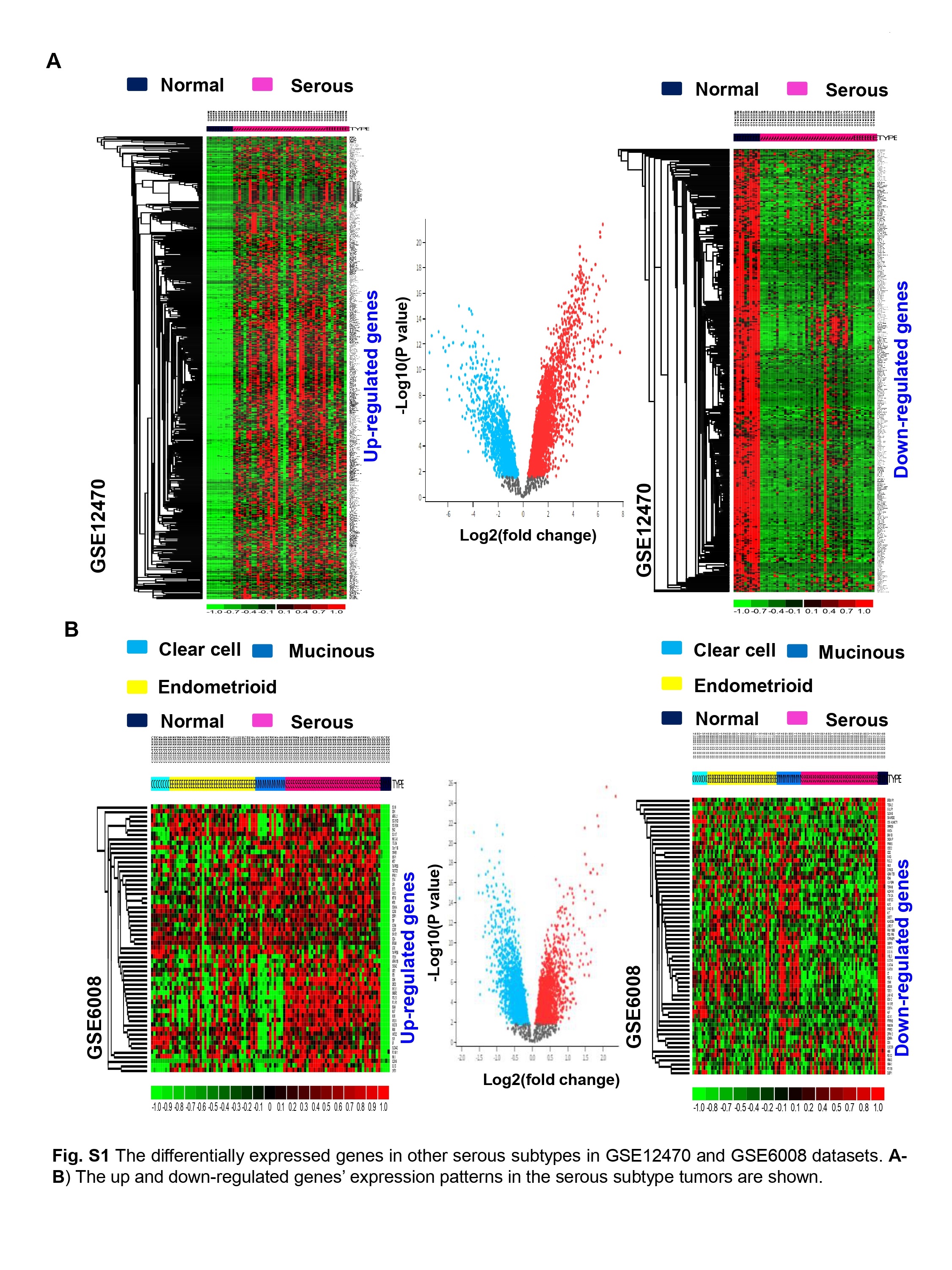


**Figure S1.** The differentially expressed genes in other serous subtypes in GSE12470 and GSE6008 datasets. (**A–B**) The up and down-regulated genes’ expression patterns in the serous subtype tumors are shown.


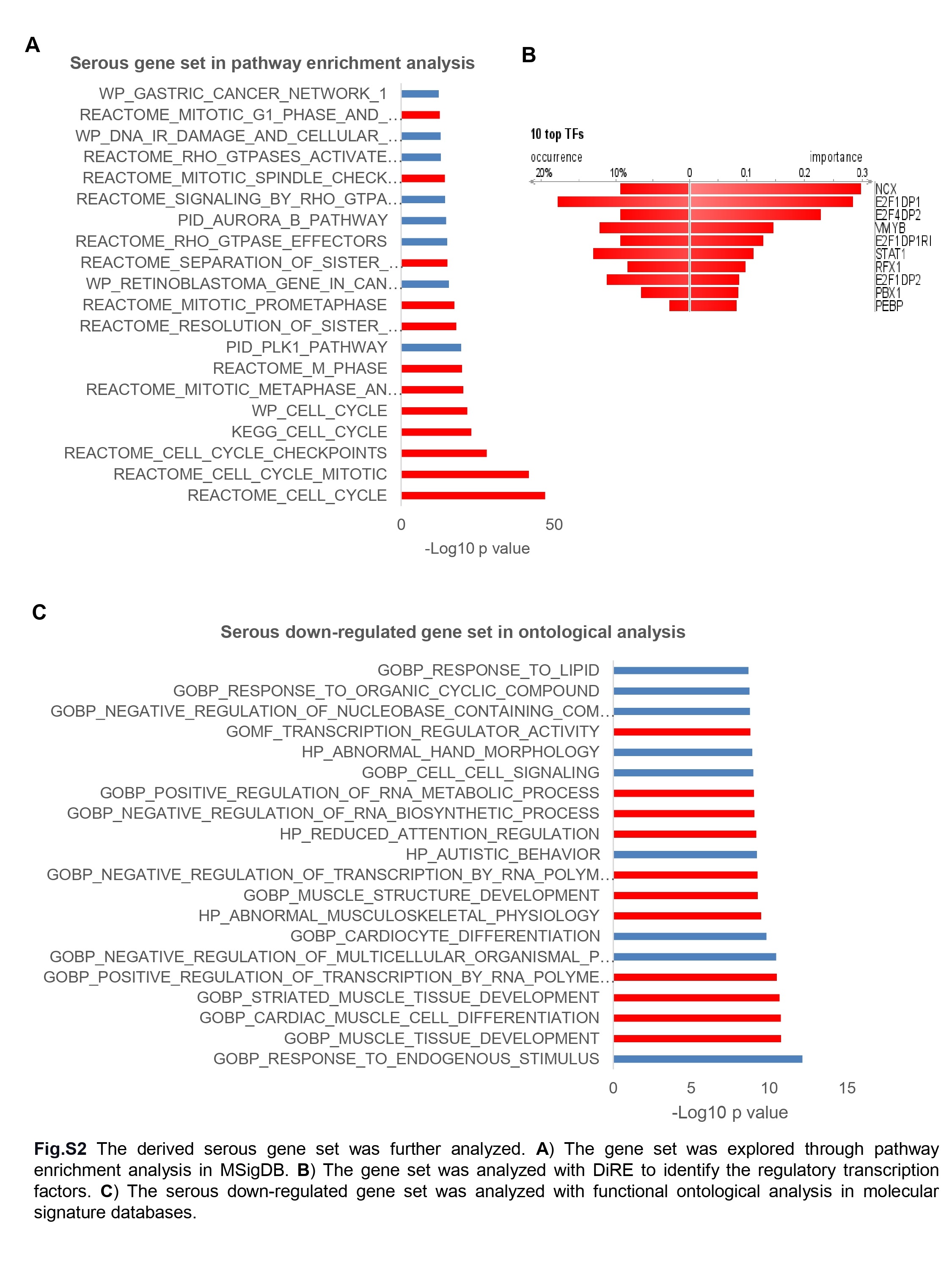


**Figure S2.** The derived serous gene set was further analyzed. (**A**) The gene set was explored through pathway enrichment analysis in MSigDB. (**B**) The gene set was analyzed with DiRE to identify the regulatory transcription factors. (**C**) The serous down-regulated gene set was analyzed with functional ontological analysis in molecular signature databases.


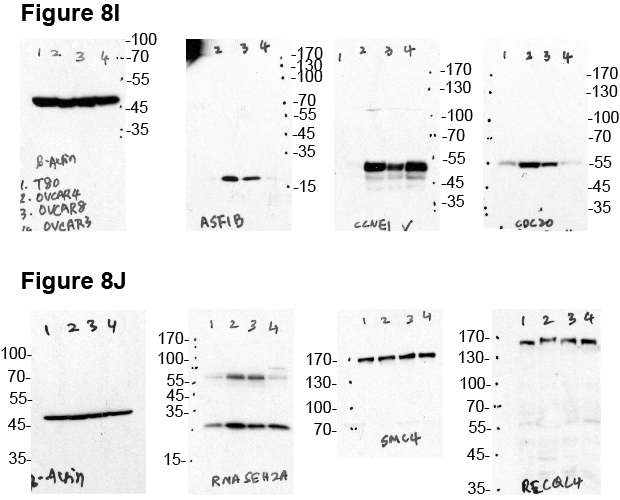


**Figure S3.** Uncropped Western Blot images from Figure 8.

**Table S1.** The list of the differentially expressed genes from the 5 datasets.

**Table S2.** The list of genes commonly upregulated in 3 or more profiles among 5 datasets used in the study.

**Table S3.** The list of genes commonly downregulated in 3 or more profiles among 5 datasets used in the study.

**Tables S1–S3.** are provided separately, attached as an Excel file.
